# Research progress on *Cornus officinalis* and its active compounds in the treatment of diabetic nephropathy

**DOI:** 10.3389/fphar.2023.1207777

**Published:** 2023-07-05

**Authors:** Chenguang Wu, Jingjing Wang, Rui Zhang, Hailing Zhao, Xin Li, Lifan Wang, Peng Liu, Ping Li

**Affiliations:** ^1^ Renal Division, Heilongjiang Academy of Chinese Medicine Sciences, Harbin, China; ^2^ China-Japan Friendship Hospital, Beijing, China; ^3^ Shunyi Hospital, Beijing Hospital of Traditional Chinese Medicine, Beijing, China

**Keywords:** diabetic nephropathy, *Cornus officinalis*, active compounds, Chinese herbal medicine, research progress

## Abstract

Diabetic nephropathy (DN) is a kidney disorder secondary to diabetes and is one of the main diabetic microvascular complications. As the number of diabetic patients grows, DN has become the leading cause of chronic kidney disease in China. Unfortunately, no definitive cure currently exists for DN. Cornus officinalis (CO), frequently utilized in clinical settings for diabetes mellitus treatment, has proven vital in both preventing and treating DN. This article explores the pathogenesis of DN and how CO and its active compounds regulate glucose and lipid metabolism, exhibit anti-inflammatory properties, inhibit oxidative stress, regulate podocytes, and manage autophagy. The mechanism and role of and its active compounds in the treatment of DN are discussed.

## Introduction

Diabetic nephropathy (DN) is one of the most common and severe complications of diabetes mellitus (DM). Associated with heightened morbidity and mortality rates among DM patients, DN is a principal cause of end-stage renal failure worldwide (Samsu, 2021). As the global prevalence of diabetes surges (Xue et al., 2017), the incidence of DN will concurrently rise unless timely and effective clinical strategies for its prevention are implemented (Gheith et al., 2016). Nowadays, Chinese herbal medicine, with its unique advantages, plays a core role in DN.

**FIGURE 1:**
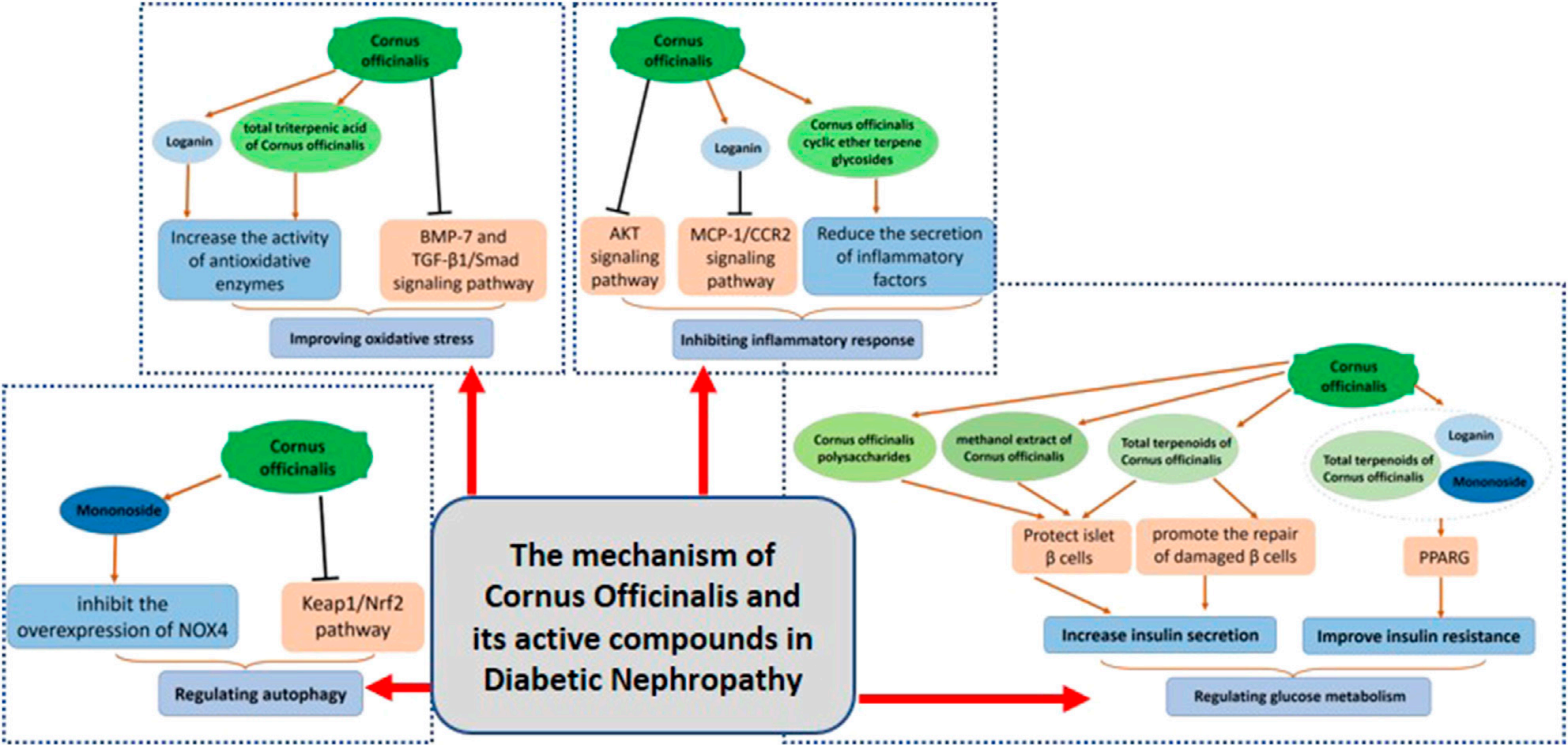
The mechanism of *Cornus Officinalis* and its active compounds in DN.

Cornus officinalis (CO) is a valuable Chinese herbal medicine (Gao et al., 2021). Modern pharmacology shows that CO has various effects such as lowering blood glucose, anti-inflammatory, antioxidant, and anti-apoptotic properties (Lou et al., 2021); and its main components, iridoid glycosides and polyphenols can significantly improve the metabolic parameters of DN (Yamabe et al., 2007a). CO effectively ameliorated insulin resistance, lowered blood glucose, and alleviated symptoms of DN patients (Park et al., 2013). Here, we illustrate the mechanism of CO and its active compounds to improve DN in Table 1 and Figure 1.

**TABLE 1:** The role of active compounds of Cornus officinalis against DN.

Mechanism	*In Vivo/In Vitro*	Model	CO extracts	References
Regulating glucose metabolism	*In Vivo*	Alloxan diabetes model mice; STZ-induced diabetes rats	Total terpenoids	Liu et al. (2012), Sharp-Tawfik et al. (2019), Samsu (2021)
		STZ-induced diabetes rats	Total saponins	Liu et al. (2012)
		Alloxan diabetes model mice; non-insulin-dependent diabetic rats	Iridoid glycoside, Loganin, Morroniside	Qian et al. (2001), Yamabe et al. (2007a), Kitada et al. (2016), Liu et al. (2016), Huang et al. (2017), Xue et al. (2017)
		STZ-induced diabetes rats	Polysaccharides	Fu et al. (2021)
Regulating lipid metabolism	*In Vivo*	STZ-induced diabetic rats	Extracts	Dzydzan et al. (2019), Gao et al. (2020)
Anti-oxidative stress	*In Vivo*	STZ-induced diabetes rats	Total triterpenoid acids	Qi et al. (2014)
Regulating autophagy	*In Vitro*	mouse podocytes	Morroniside	Gao et al. (2020)

## *Cornus officinalis* in DN

The mechanisms of CO in treating DN mainly include regulating glucose and lipid metabolism, anti-oxidative stress, anti-inflammatory, protecting podocytes, and regulating autophagy.

### Regulating glucose metabolism

Controlling blood glucose is considered an effective treatment for DN (Chatterjee et al., 2017). CO primarily lowers blood glucose through two primary pathways.

#### Increasing insulin secretion

CO total triterpenoids have no significant effect on normal mice’s blood glucose levels, but can significantly reduce the blood glucose levels of alloxan diabetic model mice, increase serum insulin levels (Han et al., 2006). The total saponins of CO can lower the blood glucose and insulin levels of streptozotocin (STZ)-induced diabetic rats (Liu et al., 2012). CO has been demonstrated to exert a protective effect on pancreatic β-cells, enhancing pancreatic pathological recovery, increasing the number of insulin-releasing β-cells, and augmenting glucose-stimulated insulin secretion (Han et al., 2014). CO extract upregulated the expression of nuclear factor of activated T-cells 2, inhibited cytokine-mediated β-cell death, improved β-cell function (Sharp-Tawfik et al., 2019). Additionally, the methanol extract of CO can reduce the expression of hepatic gluconeogenesis genes, shield pancreatic B cells from damage, and boost insulin secretion (Wu, 2012). The alcohol extraction of CO cyclic enol ether triterpenoid glycosides and administered the alcohol extract to diabetic rat models by gavage (Qian et al., 2001).

#### Enhancing insulin sensitivity and mitigating insulin resistance

CO total iridoid glycosides can effectively reduce insulin levels in mice, thus reducing blood lipids and improving insulin resistance (Liu et al., 2016).These iridoid glycosides notably improved insulin resistance in DM mice and decreased the expression levels of p-ERK1/2, p-P65, p-P38, and p-JNK1/2 proteins in liver tissues (Kitada et al., 2016). Loganin and Morroniside in CO had significant effects in improving insulin resistance in mice and promoting glucose consumption in liver cells (Huang et al., 2017). PPARG is the first gene abnormally expressed due to induced insulin resistance, playing a crucial role in insulin signaling regulation and insulin sensitivity (Malodobra-Mazur et al., 2021). CO fruit can significantly increase PPARG expression in cells, reversing insulin resistance, enhancing glucose uptake after insulin stimulation (Malodobra-Mazur et al., 2022).

Furthermore, the raw iridoid glycoside extract from CO effectively alleviated hyperglycemia symptoms and reduced the accumulation of advanced glycation end products in the kidneys (Yamabe et al., 2007b). CO total triterpenoids had a more apparent effect in inhibiting glucose absorption and promoting glucose utilization, exerting a hypoglycemic effect (Fan et al., 2017). CO polysaccharide can significantly enhance glucose uptake and consumption in IR-HepG2 cells, reduce glycated hemoglobin HbA1c levels and amylase activity, lower fasting blood glucose, and reduce body weight (Fu et al., 2021). CO extract diminishes glucose absorption by inhibiting α-glucosidase activity in intestinal villus epithelial cells. Conversely, it enhances glucose uptake and utilization by promoting insulin secretion and increasing insulin sensitivity in peripheral tissues, while also inhibiting gluconeogenesis (Gao et al., 2021).

### Regulating lipid metabolism

Lipid peroxidation is a significant contributor to the damage of blood cell structures in diabetic patients. Studies have shown that CO fruit extract can raise the levels of GSH and MCH in diabetic rats, and the antioxidants, iridoid glycosides. The polyphenols in the extract can neutralize active oxygen forms and inhibit lipid peroxidation, thus providing antioxidative protection (Dzydzan et al., 2019). CO extract can also lower blood lipid levels in STZ-induced diabetic rats (Gao et al., 2020).

### Improving oxidative stress

Oxidative stress is a state in which tissue damage occurs due to an imbalance between oxidants and antioxidants (Kao et al., 2010). CO extract has significant antioxidative activity and increases the activity of antioxidative enzymes, with strong scavenging abilities for ABTS free radicals (Hwang et al., 2016). CO can reduce the kidney weight ratio, downregulate plasma creatinine, urea nitrogen, and uric acid levels, modulate the BMP-7 and TGF-β1/Smad signaling pathway, downregulate ROS, MCP-1, NF-κB expression (Li et al., 2015). Loganin can lower reactive oxygen levels, improve leukocyte antioxidative status, boost activities of glutathione reductase, glutathione peroxidase, and catalase, thereby increasing GSH levels in leukocytes (Liu et al., 2012). Despite total triterpenic acid from CO not significantly impacting blood glucose levels in diabetic rats, it can reduce urinary protein, Scr, and BUN levels and improve renal pathological changes. Total triterpenic acid can decrease MDA levels and augment superoxide dismutase, catalase, and glutathione peroxidase activities, reducing the expression of TGF-β1 in the kidney tissue of diabetic rats (Qi et al., 2014).

### Anti-inflammatory

Immune and inflammatory responses play an important role in the pathogenesis of DN (Tavafi, 2013). Numerous studies suggest that IL-1β, IL-6, and IL-18 are major contributors to the progression of diabetic nephropathy (Wong et al., 2007). Experimental findings reveal that CO extract can disrupt LPS-induced macrophages and reduce the expression of IL-1β, IL-6, and NF-κB. Its renoprotective effect may be realized by downregulating AKT phosphorylation (Najjar et al., 2021). Loganin significantly inhibit macrophage infiltration, and counter inflammation, suggesting that Loganin’s therapeutic effect on DN may be related to inhibiting macrophage infiltration, activating the MCP-1/CCR2 signaling pathway, and suppressing inflammatory cytokines (Du et al., 2021). CO cyclic ether terpene glycosides significantly inhibit the secretion levels of AGEs-induced inflammatory factors, and dose-dependently alleviate the AGEs-induced inflammation in rat mesangial cells HBZY-1 (Guo et al., 2013).

### Protected podocytes

Morroniside has a protective effect on podocyte apoptosis, and its mechanism may involve restoring blocked autophagy flux and inhibiting H_2_O_2_-induced NADPH oxidase 4 (NOX4) overexpression to prevent podocyte apoptosis (Gao et al., 2020). The optimal compatibility of components in CO has been shown to protect against early kidney disease in type 2 diabetic rats by increasing WT1 expression in glomerular podocytes (Liu et al., 2012). Loganin and morroniside may jointly inhibit the apoptosis of podocytes in DN by targeting AGEs RAGE and its downstream pathways p38 MAPK and Nox4 (Chen et al., 2020).

### Regulating autophagy

The main clinical pathological changes in DN are glomerulosclerosis and renal tubulointerstitial fibrosis, with renal interstitial fibrosis characterized by renal parenchymal cell injury and massive extracellular matrix deposition (Guo et al., 2022). CO can promote a highly protective mechanism in pancreatic β-cells through autophagy and the Kelch-like ECH-associated protein 1 (Keap1)/nuclear factor erythroid 2-related factor 2 (Nrf2) pathway mediated by the p62 adapter molecule (Sharp-Tawfik et al., 2022). Morroniside can reduce oxidative stress-induced podocyte apoptosis by restoring impaired autophagic flux and inhibiting the overexpression of NOX4 (Gao et al., 2020).

### Others

CO can improve kidney injury in DN rats and ultimately exert kidney-protective effects by blocking the activation of the Wnt/β-catenin signaling pathway and regulating the composition of gut microbiota (Ju et al., 2022). Treatment with CO significantly improved the intestinal microbiota composition and fecal metabolite characteristics in CKD rats, and CO can reverse the CKD condition (Zhang et al., 2021).

### Traditional Chinese medicine prescription and clinical trials containing CO

Common traditional Chinese medicine prescriptions containing cornus officinalis for the clinical treatment of DKD include Shenqi Dihuang Decoction, Jinkui Shenqi Pill, and Liuwei Dihuang Pill. Experiments have shown that Shenqi Dihuang Decoction can intervene in the inflammatory response of early DN patients, reduce proteinuria, protect renal function, and improve the patient’s endothelial function and hemorheology, achieving normal recovery of microcirculation (Wang et al., 2018). Studies by Liu Chunyan and others found that Jinkui Shenqi Pill can downregulate the phosphorylation levels of JNK1 and Bcl-2, significantly reduce the fragments of activated caspase-3, and may protect podocytes and delay the progress of DKD through the JNK1/Bcl-2 pathway (Liu et al., 2019). Liuwei Dihuang decoction has been proven to alleviate early DN abnormalities mediated by the inhibition of the endothelin-1 reactive oxygen species (ET-ROS) system and increasing MMPs activity (He et al., 2007). In the study of medication rules, Cornus officinalis appears with a frequency of >30%. Its nourishing and kidney-replenishing effects can cope with the basic pathogenesis of both Qi and Yin deficiency in DN (Wen et al., 2015). In the clinical information system analysis summary of traditional Chinese medicine treatment of diabetic nephropathy medication rules by Yang Chaomao and others, Cornus officinalis was ranked in the top 5 in terms of usage frequency. There is a strong correlation between the three medicines Salvia miltiorrhiza, Cornus officinalis, and Astragalus. Combined use can strengthen the spleen, nourish the livers and kidneys, and replenish Qi, nourish blood and remove blood stasis (Yang et al., 2022).

## Conclusion and perspectives

The pathogenesis of DN is complex, and there is less research on the role of CO in autophagy and the regulation of gut microbiota. More in-depth investigations should be conducted on additional mechanisms. Chinese herbal medicine has complex ingredients, and more effective components of CO can be explored. The therapeutic effect of CO in improving inflammation and oxidative stress of glomerular mesangial cells, podocytes and renal tubular epithelial cells in DN is gradually becoming clear. Moving forward, the treatment of DN with CO can be discussed from multiple angles, levels, and directions, with the aim of providing better treatment for patients.
